# Supplementary material for: Environmental deformations dynamically shift the grid cell spatial metric
Source: eLife. 2018 Oct 22;7:e38169. doi: 10.7554/eLife.38169 (PMC6203432; doi:10.7554/eLife.38169)
Supplement: Transparent reporting form [file elife-38169-transrepform.pdf]

1st Floor
24 Hills Road
Cambridge CB2 1JP, UK

P 01223 855340
W elifesciences.org
T @elife

eLife's transparent reporting form

We encourage authors to provide detailed information *within their submission* to facilitate the interpretation and replication of experiments. Authors can upload supporting documentation to indicate the use of appropriate reporting guidelines for health-related research (see EQUATOR Network), life science research (see the BioSharing Information Resource), or the ARRIVE guidelines for reporting work involving animal research. Where applicable, authors should refer to any relevant reporting standards documents in this form.

If you have any questions, please consult our Journal Policies and/or contact us: editorial@elifesciences.org.

Sample-size estimation

- You should state whether an appropriate sample size was computed when the study was being designed
- You should state the statistical method of sample size computation and any required assumptions
- If no explicit power analysis was used, you should describe how you decided what sample (replicate) size (number) to use

Please outline where this information can be found within the submission (e.g., sections or figure legends), or explain why this information doesn't apply to your submission:

Where relevant for simulations, sample size is described in the accompanying figure caption (Figure 5). For reanalysis of the two recorded grid cell rescaling datasets, we were limited to the sample sizes previously recorded and report how units were selected in our Materials and Methods. However, the sample sizes of both datasets were sufficient to observe related rescaling effects within each dataset independently; thus when combined we expect sufficient power to address our questions of interest.

Replicates

- You should report how often each experiment was performed
- You should include a definition of biological versus technical replication
- The data obtained should be provided and sufficient information should be provided to indicate the number of independent biological and/or technical replicates
- If you encountered any outliers, you should describe how these were handled
- Criteria for exclusion/inclusion of data should be clearly stated
- High-throughput sequence data should be uploaded before submission, with a private link for reviewers provided (these are available from both GEO and ArrayExpress)

Please outline where this information can be found within the submission (e.g., sections or figure legends), or explain why this information doesn't apply to your submission:

eLife Sciences Publications, Ltd is a limited liability non-profit non-stock corporation incorporated in the State of Delaware, USA, with company number 5030732, and is registered in the UK with company number FC030576 and branch number BR015634 at the address 1st Floor, 24 Hills Road, Cambridge CB2 1JP | August 2014

Each simulation yielded multiple grid and place units as described in the Results section *A model of border, grid, and place cell interactions* and the Materials and Methods section *Unit sampling*. Sampling of grid units for Figure 5 and related supplements is also reported in the caption for that figure. Simulation data and code for running additional simulation replicates are available on Github, as noted in the *Data and code availability* section of the Materials and Methods. For recorded data, grid cell counts from each experiment and manipulation are described in the first paragraph of the Results section *Boundary-tethered shifts in grid phase are observed in recorded grid cells during rescaling deformations*. All cells from the Stensola et al. (2012) dataset were included – these cells previously met sufficient criteria to be classified as grid cells as described in the original publication. The Barry et al. (2007) dataset included both grid and non-grid cells recorded in the medial entorhinal cortex. To select grid cells from these data we used the criteria described in the Materials and Methods section *Reanalysis of experimental data*. Data were typically combined across experiments for analysis in the Results section *Boundary-tethered shifts in grid phase are observed in recorded grid cells during rescaling deformations*, with source data files containing the results of our reanalyses. In some cases, only a theoretically-motivated subset of manipulations were included; these cases are clearly described in the caption for Figures 2 and 3, as well as the related paragraphs of the Results section *Boundary-tethered shifts in grid phase are observed in recorded grid cells during rescaling deformations*.

eLIFE
1st Floor
24 Hills Road
Cambridge CB2 1JP, UK
P 01223 855340
W elifesciences.org
T @elife

Statistical reporting

- Statistical analysis methods should be described and justified
- Raw data should be presented in figures whenever informative to do so (typically when N per group is less than 10)
- For each experiment, you should identify the statistical tests used, exact values of N, definitions of center, methods of multiple test correction, and dispersion and precision measures (e.g., mean, median, SD, SEM, confidence intervals; and, for the major substantive results, a measure of effect size (e.g., Pearson's r, Cohen's d)
- Report exact p-values wherever possible alongside the summary statistics and 95% confidence intervals. These should be reported for all key questions and not only when the p-value is less than 0.05.

Please outline where this information can be found within the submission (e.g., sections or figure legends), or explain why this information doesn't apply to your submission:

Each statistical test is noted accompanying the reported statistic. All tests are 2-tailed unless stated otherwise, and all error bars denote ± 1 standard error of the mean as described in the Materials and Methods section *Statistical tests*. Exact p-values, summary statistic values, and degrees of freedom are provided within the text or in the related figure caption (Fig. 6) for each test. All reanalyses also include source data files.

(For large datasets, or papers with a very large number of statistical tests, you may upload a single table file with tests, Ns, etc., with reference to sections in the manuscript.)

Group allocation

- Indicate how samples were allocated into experimental groups (in the case of clinical studies, please specify allocation to treatment method); if randomization was used, please also state if restricted randomization was applied
- Indicate if masking was used during group allocation, data collection and/or data analysis

Please outline where this information can be found within the submission (e.g., sections or figure legends), or explain why this information doesn't apply to your submission:

This information does not apply to our submission as samples were not allocated into different experimental groups.

Additional data files ("source data")

- We encourage you to upload relevant additional data files, such as numerical data that are represented as a graph in a figure, or as a summary table
- Where provided, these should be in the most useful format, and they can be uploaded as "Source data" files linked to a main figure or table
- Include model definition files including the full list of parameters used
- Include code used for data analysis (e.g., R, MatLab)
- Avoid stating that data files are "available upon request"

Please indicate the figures or tables for which source data files have been provided:

Simulation code and results are available on Github, as noted in the *Data and code availability* section of the Materials and Methods. As our reanalyses rely on previously published data that are owned by other labs, we are not authorized to upload the raw data files; however, all values generated during our reanalysis are included as source data files.

All original reanalyzed data were originally reported in the following papers:

1) Barry et al., 2007. Experience-dependent rescaling of entorhinal grids. https://doi.org/10.1038/nn1905

2) Stensola et al., 2012. The entorhinal map is descritized. https://doi.org/10.1038/nature11649

These data are available upon request from the corresponding authors of these papers.

eLife Sciences Publications, Ltd is a limited liability non-profit non-stock corporation incorporated in the State of Delaware, USA, with company number 5030732, and is registered in the UK with company number FC030576 and branch number BR015634 at the address 1st Floor, 24 Hills Road, Cambridge CB2 1JP | August 2014
